# Accuracy of the Raja Isteri Pengiran Anak Saleha Appendicitis (RIPASA) and Alvarado Score for Acute Appendicitis: A Comparative Study

**DOI:** 10.7759/cureus.93756

**Published:** 2025-10-03

**Authors:** Zakia Sultana, Gohar Ali, Haq Nawaz, Jawad Ali, Anees Ahmed, Aiman Ali, Muhammad Tahir, Yousaf Jan

**Affiliations:** 1 Department of General Surgery, Hayatabad Medical Complex Peshawar, Peshawar, PAK; 2 Department of Orthopedic Surgery, Khyber Teaching Hospital, Peshawar, PAK; 3 General Surgery, Naas General Hospital, Naas east, IRL

**Keywords:** acute appendicitis, alverado score, diagnosis of acute appendicitis, diagnostic accuracy analysis, ripasa score

## Abstract

Background: Acute appendicitis is the most frequent surgical emergency worldwide, but due to atypical symptoms, its diagnosis is challenging. The Alverado score is widely used but is limited in Asian populations, prompting the development of the Raja Isteri Pengiran Anak Saleha Appendicitis (RIPASA) score. This study aims to compare the accuracy of both systems.

Methodology: A prospective study was conducted at Hayatabad Medical Complex, Peshawar, from August 2024 to September 2025, including 100 patients with suspected acute appendicitis. Both RIPASA and Alvarado scores were assessed and compared with intraoperative findings using the Gomez classification. Sensitivity, specificity, positive predictive value (PPV), negative predictive value (NPV), and overall diagnostic accuracy were calculated.

Results: The mean patient age was 28.1 years; 54% were male. Common presenting features were anorexia (90%), tenderness (99%), rebound tenderness (94%), and pain migration (76%). The RIPASA score showed sensitivity of 91.9%, specificity of 64.3%, PPV of 94.1%, NPV of 56.3%, and accuracy 88%. The Alvarado score demonstrated sensitivity of 94.2%, specificity of 57.1%, PPV of 93.1%, NPV of 61.5%, and accuracy 89%. Receiver operating characteristic (ROC) analysis yielded an area under the curve (AUC) of 0.78 for RIPASA and 0.76 for Alvarado.

Conclusion: Both the RIPASA and Alvarado scores demonstrated high accuracy, with Alvarado showing slightly higher sensitivity (94.2% vs. 91.9%) and RIPASA offering better specificity (64.3% vs. 57.1%). Overall, both scoring systems proved reliable tools for diagnosis, helping reduce negative appendectomy rates and improving clinical decision-making.

## Introduction

Acute appendicitis affects one in every seven people worldwide, often causing significant right lower abdominal pain and peak incidence in young adults [1,2]. It is a frequent surgical emergency that requires prompt intervention to avoid serious complications or death [2-4]. Acute appendicitis is most often caused by obstruction of the appendix, usually by a faecolith, leading to increased intraluminal pressure, ischemia, bacterial overgrowth, and inflammation that can progress to perforation if untreated [5]. Diagnosing acute appendicitis is challenging due to atypical symptoms that mimic other conditions, with only 40% of cases showing classic signs like periumbilical pain, nausea, vomiting, cough, and pain migrating to the right lower quadrant [2,6,7]. Several conditions, including carcinoid tumors, mucocele, diverticula, and appendiceal intussusception, can mimic the presentation of acute appendicitis [5,8]. Despite advances in modern imaging and laboratory investigations, the diagnosis of acute appendicitis remains a clinical challenge, largely due to atypical presentations, limited resources in certain settings, and the risk of unnecessary negative appendectomies, thereby continuing to rely heavily on clinical and surgical judgment. [6,8,9]. Non-operative management with antibiotics may be considered in carefully selected patients with uncomplicated appendicitis, whereas surgery remains the standard treatment in most cases. The updated consensus of Jerusalem guidelines 2020 shows various aspects of acute appendicitis [6].

Several scoring systems have been developed over the past few decades to aid diagnosis and reduce the negative appendectomy rate [1,10,11]. The Alfredo Alvarado score, introduced in 1986, is based on six parameters [1,12]. Scores of 4-5 suggest possible appendicitis, 7-8 indicate probable appendicitis, and 9-10 indicate a high likelihood, but the score is not very specific in the literature [13]. The Alvarado score was found to be inadequate for diagnosing appendicitis in Asian and Middle Eastern populations, leading to the development of the Raja Isteri Pengiran Anak Saleha Appendicitis (RIPASA) score, which is better suited for these groups [11,13]. The RIPASA score was more accurate in identifying patients with acute appendicitis and correctly classified patients without the condition [1,11,13].

Studies found that the RIPASA score had higher sensitivity and specificity compared to the Alvarado score, but in contrast, other studies revealed that very few patients had a normal appendix upon histological evaluation [3,14]. The RIPASA score accurately identified most patients with acute appendicitis and had a lower negative appendectomy rate compared to the Alvarado score [3].

This study aims to compare the diagnostic accuracy of these two scoring systems in a cohort of patients presenting with suspected acute appendicitis, to determine the more reliable clinical score for decision-making in acute appendicitis, and to reduce the unnecessary negative appendectomy in resource-limited hospitals.

## Materials and methods

Study design and settings

This prospective observational study was conducted in Hayatabad Medical Complex, Peshawar, from 1 August 2024 to 1 September 2025. The study was approved by the Ethical Review Board of Hayatabad Medical Complex, Peshawar (HMC-QAD-F-2019) and conducted according to the Declaration of Helsinki and Strengthening the Reporting of Cohort Studies in Surgery guidelines (STROCSS) [15].

Sample

A total of 100 patients were included in this study following exclusion criteria out of 113, 13 cases were excluded (113 total), which was estimated using the WHO calculator [16] with a 95% confidence interval and a 5% margin of error.

The formula n=Z2×p×(1−p)/d2 is used.

Where: n = required sample size, Z = Z-score for desired confidence level, p= expected prevalence (proportion), d = desired margin of error (absolute precision).

Inclusion and exclusion criteria

All patients who are clinically suspicious of having acute appendicitis, supported by ultrasonography and blood tests, with findings suggestive of appendicitis, are included. Patients who underwent both RIPASA and Alvarado scoring were performed by the same surgical team. Patients younger than 15 years of age, pregnant patients, patients with an appendicular mass, and patients presenting with generalized peritonitis or peritonitis-like symptoms are excluded from the study.

Data collection and outcome measures

After taking informed consent from all the patients, data were collected under the standardized proforma, which includes variables of documenting medical history, comorbidities, demographics, examination findings, laboratory and imaging results, and postoperative details according to both the score variables.

Statistical analysis

Collected data was entered into SPSS 23.0v and analyzed. Quantitative data were presented as frequencies and percentages. Sensitivity and specificity of both the scores were assessed by using the Gomez classification based on visual intraoperative grades of appendicitis. The patients who have low values in both scores, simultaneously with a low Gomez score, were considered as negative appendicitis. The chi-square test was used for comparing the categorical variables, and the results were tabulated. Sensitivity, specificity, positive predictive value (PPV), negative predictive value (NPV), and diagnostic accuracy of both scores were calculated. A p-value of 0.05 or lower was considered significant.

## Results

Tables 1-2 and Table 4 present the Alvarado and RIPASA scoring parameters, with positive scores defined as ≥ 7 for Alvarado and ≥ 12 for RIPASA. The mean age of patients was 28.1 ± 11.7 years with a positively skewed distribution (skewness 1.21), indicating most patients are in their early to mid-20s. The majority (79%) are younger than 40 years, as shown in Table 3. 54% of cases are male, while female 46%. The most common presenting signs and symptoms are loss of appetite (90%), tenderness (99%), rebound tenderness (94%), and pain migration to the right lower quadrant (76%). Nausea or vomiting is present in 50%, guarding in 29%, and pain in the right iliac fossa on left iliac fossa compression in 13% of patients (Table 3).

**Table 1:** Alvarado Score This table lists the clinical and laboratory criteria used in the Alvarado scoring system, along with the corresponding weightage for each parameter.

Alvarado	Score
Migratory pain	1
Anorexia	1
Nausea	1
Tenderness in the right iliac fossa	2
Rebound tenderness	1
Elevated temperature	1
Raised WBC count	2
Shift to left (neutrophil shift)	1
Total Score	10

**Table 2:** RIPASA Score This table describes the clinical and demographic variables used in the RIPASA scoring system. Each parameter is assigned a specific score, with a total score ≥ 12 considered positive for acute appendicitis. RIPASA: Raja Isteri Pengiran Anak Saleha Appendicitis

RIPASA	Score		Score
Gender		Clinical Signs	
Male	1.0	Tenderness in right iliac fossa	1.0
Female	0.5	Guarding in right iliac fossa	2.0
Age		Rebound tenderness	1.0
< 40 years	1.0	Rovsing’s sign	2.0
> 40 years	0.5	Elevated temperature	1.0
Symptoms		Investigations	
Pain in right iliac fossa	0.5	Raised WBC count	1.0
Migratory pain	0.5	Unremarkable urinalysis	1.0
Anorexia	1.0	Demographics	
Nausea/Vomiting	1.0	Foreign nationality	1.0
Length of symptoms < 48 hours	1.0	Total Score	17.5
Length of symptoms > 48 hours	0.5		

**Table 3:** Descriptive Preoperative clinical features. This table shows patient demographics (n=100) and presenting symptoms. Most patients were <40 years, with a nearly equal gender distribution. Common features included loss of appetite, pain migration, RIF pain, and tenderness, while guarding and Rovsing’s sign were less frequent.

Characteristic		Frequency (n)	Percentage (%)
Age	<40	79	79
	>40	21	21
Gender	Male	54	54
	Female	46	46
Symptoms and signs			
Pain in the right iliac fossa (RIF)	Yes	55	55.0
	No	45	45.0
Pain migration to right lower quadrant	Yes	76	76.0
	No	24	24.0
Loss of appetite	Yes	90	90.0
	No	10	10.0
Presence of nausea or vomiting	Yes	50	50.0
	No	43	43.0
Presence of tenderness	Yes	99	99.0
	No	1	1.0
Presence of rebound tenderness	Yes	94	94.0
	No	4	4.0
Guarding in the right iliac fossa	Yes	29	29.0
	No	71	71.0
Pain elicited in RIF by compressing LIF	Yes	13	13.0
	No	87	87.0
Duration of symptoms< 48hrs	Yes No	45 55	45% 55%

**Table 4:** Normal Ranges of RIPASA and Alvarado Scores This table summarizes the frequency of patients categorized above and below the diagnostic cut-off values (≥7 for Alvarado, ≥12 for RIPASA). RIPASA: Raja Isteri Pengiran Anak Saleha Appendicitis

Participants Scores		Frequency
Alvarado score	≥7	85
	<7	15
RIPASA score	≥ 12	81
	<12	19

**Figure 1:**
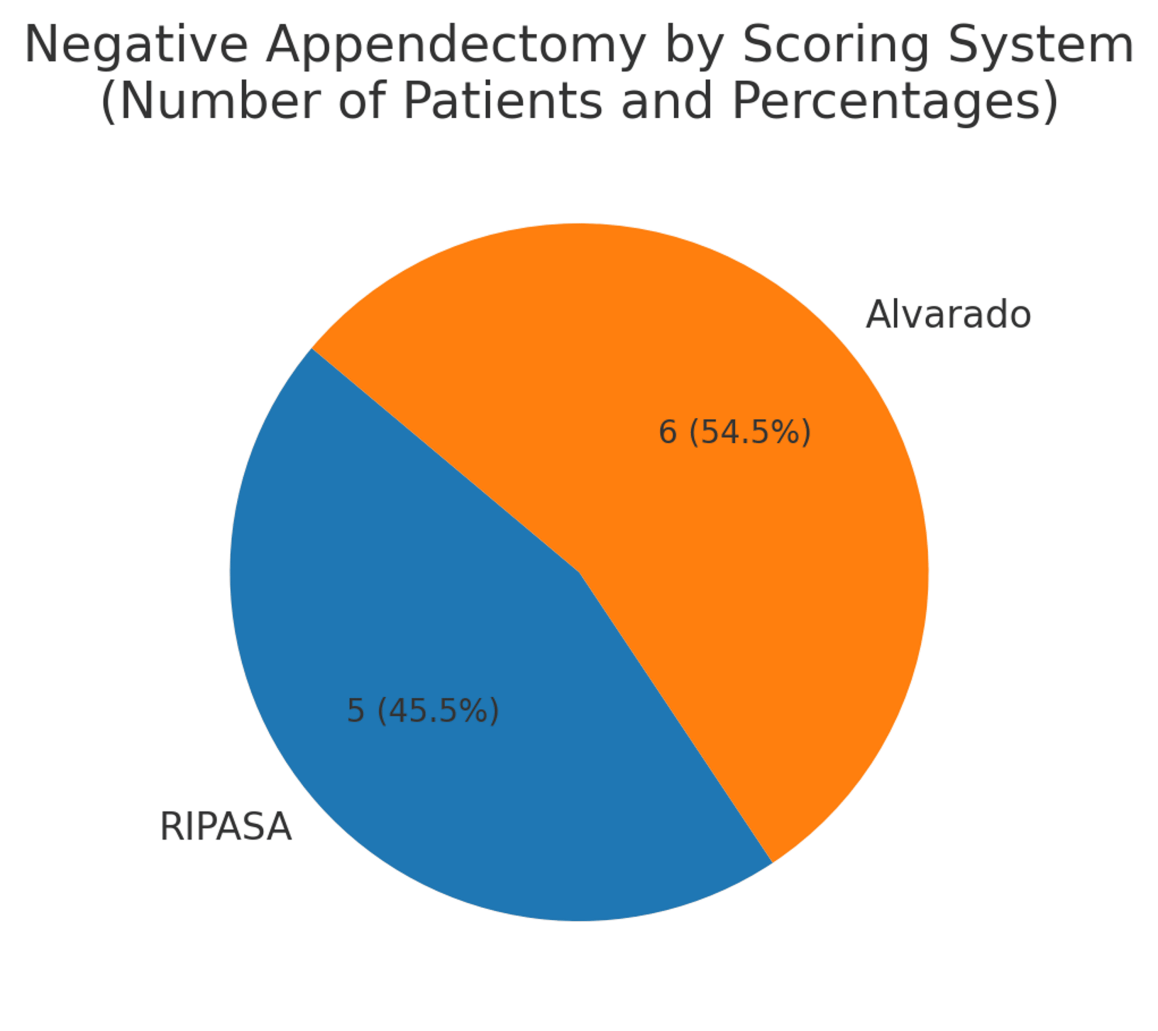
Negative appendectomy rate. This pie chart illustrates the distribution of negative appendectomy cases based on the Alvarado and RIPASA scoring systems. Out of 11 patients with negative appendectomies, 6 (54.5%) were identified using the Alvarado score, while 5 (45.5%) were identified using the RIPASA score. The figure highlights the relative proportion of false-positive diagnoses associated with each scoring system in this study. RIPASA: Raja Isteri Pengiran Anak Saleha Appendicitis

The Alvarado score is ≥7 in 85% of patients, while RIPASA is ≥12 in 81% shown in Table 4. Negative appendectomy rates are five cases with RIPASA and six cases with Alvarado scoring. As shown in Figure 1. Both the scores have some negative appendectomy rate, which was decided by using the Gomez score system, shown in Figure 1.

In Table 5, out of the total 100 patients, 84 had a RIPASA score ≥12, of whom 79 were confirmed to have acute appendicitis intraoperatively. The diagnostic value of the RIPASA score showed a sensitivity of 91.86%, specificity of 64.29%, PPV of 94.05%, NPV of 56.25%, and an overall diagnostic accuracy of 88% (p ≤ 0.05).

**Table 5:** A) Cross-tabulation of RIPASA Score with intraoperative findings, B) Diagnostic performance of RIPASA Score. Table Part A presents the cross-tabulation of diagnostic scores with intraoperative findings; Part B shows the Diagnostic performance of the RIPASA Score. *Note: p-value < 0.001 is considered statistically significant PPV: Positive Predictive Value, NPV: Negative Predictive Value; CI: Confidence interval; RIPASA: Raja Isteri Pengiran Anak Saleha Appendicitis

RIPASA	Finding Appendicitis Intraoperatively (Gomez)		Total
Part A.	Positive	Negative	
≥7.5	79	5	84
<7.5	7	9	16
	86	14	100
Part B.	Estimate (%)	p-value*	95% CI
Sensitivity	91.86%	< 0.001	84.1–96.0%
Specificity	64.29%	< 0.001	38.8–83.7%
PPV	94.05%	< 0.001	86.8–97.4%
NPV	56.25%	< 0.001	33.2–76.9%
Diagnostic Accuracy	88.00%		

Similarly, in Table 6, out of 100 patients, 87 had an Alvarado score ≥7, of whom 81 were confirmed to have appendicitis intraoperatively. The Alvarado score demonstrates a sensitivity of 94.19%, specificity of 57.14%, PPV of 93.10%, NPV of 61.54%, and an overall diagnostic accuracy of 89%.

**Table 6:** Cross-tabulation of Alvarado Score with intraoperative findings Part A shows the cross-tabulation of the Alvarado score with intraoperative findings. Part B shows diagnostic performance parameters of the Alvarado Score. *Note: p-value p < 0.001 is considered statistically significant PPV: Positive Predictive Value, NPV: Negative Predictive Value, CI: Confidence Interval

Alvarado Score	Finding Appendicitis Intraoperatively (Gomez)		Total
Part A	Positive	Negative	
≥7	81	6	87
<7	5	8	13
	86	14	100
Part B	Estimate (%)	p-value*	95% CI
Sensitivity	94.19%	<0.001	87.1–97.5%
Specificity	57.14%	<0.001	32.6–78.6%
PPV	93.10%	<0.001	85.8–96.8%
NPV	61.54%	<0.001	35.5–82.3%
Diagnostic Accuracy	89.00%		

**Figure 2:**
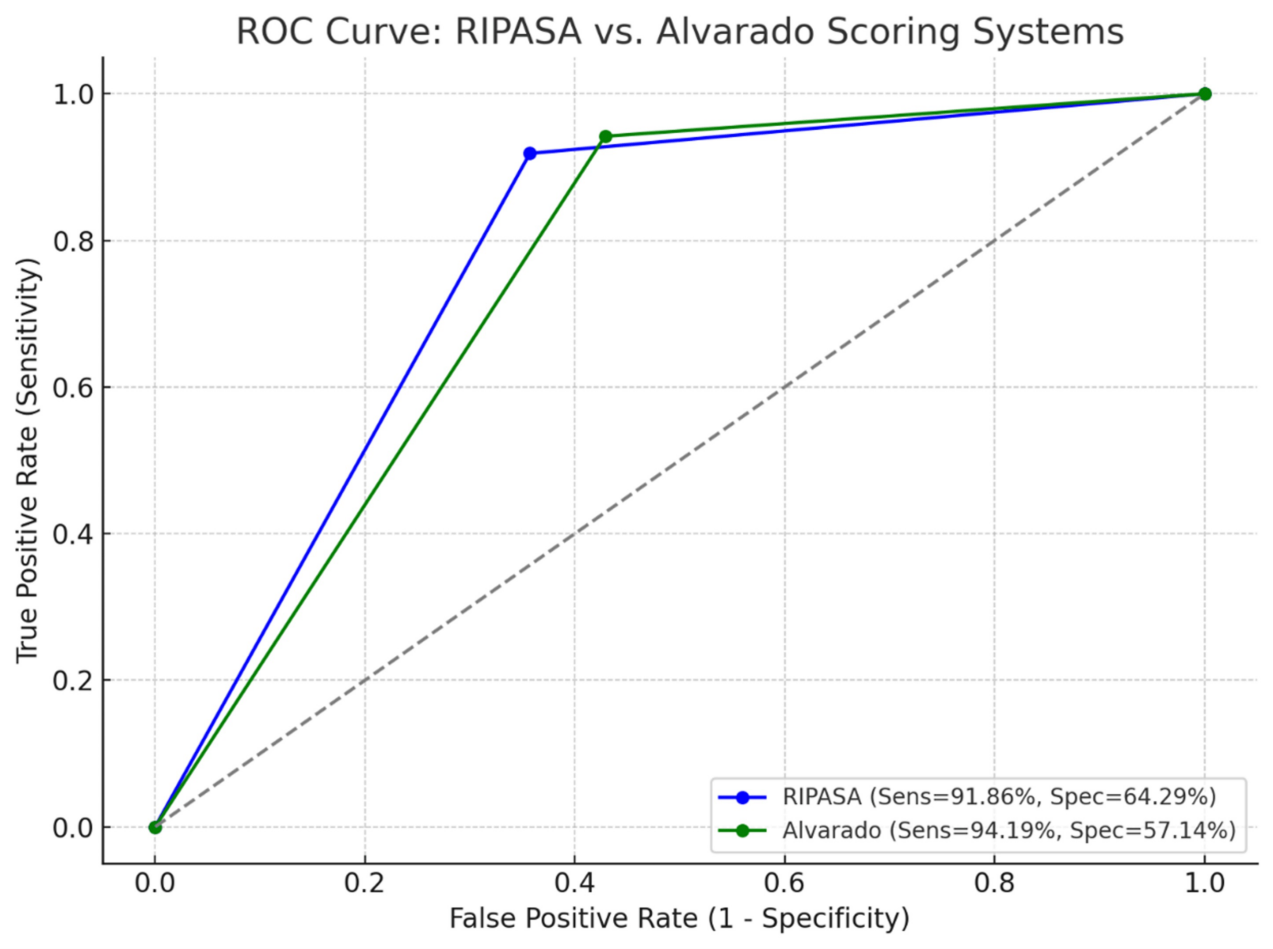
ROC curve comparing the RIPASA and Alvarado scoring systems. This figure shows the receiver operating characteristic (ROC) curves comparing the diagnostic performance of the RIPASA and Alvarado scoring systems in predicting acute appendicitis. The RIPASA score demonstrated a sensitivity of 91.86% and specificity of 64.29%, while the Alvarado score showed a sensitivity of 94.19% and specificity of 57.14%. The ROC analysis illustrates that both scoring systems achieved high sensitivity, with RIPASA demonstrating slightly higher specificity in this cohort. RIPASA: AUC ≈ 0.90 (95% CI: 0.83–0.96); Alvarado: AUC ≈ 0.89 (95% CI: 0.82–0.95) RIPASA: Raja Isteri Pengiran Anak Saleha Appendicitis

In Figure 2, the receiver operating characteristic (ROC) curves for both the RIPASA and Alvarado scoring systems were analyzed. The RIPASA (Blue line) score demonstrated a sensitivity of 91.86% and specificity of 64.29%, while the Alvarado score (Green line) showed a sensitivity of 94.19% and specificity of 57.14% at their respective optimal cut-off thresholds. The area under the curve (AUC) was 0.78 for RIPASA and 0.76 for the Alvarado score. These results show strong diagnostic performance for both systems, with RIPASA showing slightly better specificity than Alvarado. In conclusion, comparing both scores, RIPASA shows slightly better specificity, with Alvarado marginally higher sensitivity. ROC analysis confirmed the strong performance of both systems, with negative appendectomy rates remaining low and comparable. Overall, both scores are reliable diagnostic tools, with RIPASA offering a statistical advantage in identifying true negative cases.

## Discussion

Acute appendicitis is the most common acute abdominal condition, which is a surgical emergency worldwide [2,4]. Appendicitis requires prompt appendectomy because if untreated, an inflamed appendix will eventually perforate and spill infectious material into the abdominal cavity. This can cause peritonitis, which has poor morbidity and even mortality [2,6]. The diagnosis of acute appendicitis is complicated due to its vague and unusual symptoms [7,16,17]. In most cases, appendicitis can be diagnosed clinically, with tests like inflammatory markers and ultrasound helping when it’s unclear, and treatment is appendectomy after giving fluids, pain relief, and antibiotics [6,17]. Therefore, developing different scores helps to evaluate and ease the diagnosis of acute appendicitis. In our study, we compare the diagnostic accuracy of the Alvarado and RIPASA scoring systems in patients with suspected acute appendicitis [3,9,14,16,18,19]. Both scores demonstrated high sensitivity and specificity, confirming their value as diagnostic tools [6,7,20]. The Alvarado score showed slightly higher sensitivity and NPV, while the RIPASA score had better specificity and PPV. Overall diagnostic accuracy was comparable (89% vs. 88%), with Alvarado having a slight edge. Our study has similar results to those of Shams et al. RIPASA score for Asian people [10]. In our study, the RIPASA score showed a sensitivity of 91.86%, specificity of 64.29%, PPV of 94.05%, NPV of 56.25%, and an overall diagnostic accuracy of 88%. The Alvarado score demonstrated a sensitivity of 94.19%, specificity of 57.14%, PPV of 93.10%, NPV of 61.54%, and diagnostic accuracy of 89%. These results indicate that both scoring systems performed similarly in diagnosing acute appendicitis, with the Alvarado score showing slightly higher sensitivity and overall accuracy, while RIPASA had marginally better specificity and PPV. This study is consistent with the findings of Shams et al. and Chong et al. [3,10].

These findings are consistent with literature like Chisthi et al., Shams et al., Mumtaz et al., and Chong et al. reporting high diagnostic accuracy of both scoring systems in different populations [1,3,10,19]. Our results suggest that structured scoring helps reduce diagnostic uncertainty, guides rational use of imaging, and may lower negative appendectomy rates. As mentioned by Mumtaz et al. and Chong et al. The RIPASA score has lowered the negative appendectomy rate, as in our study.

Alvarado showed slightly higher sensitivity and NPV, while RIPASA performed better in terms of specificity and PPV. Overall, both scores were statistically significant and effective diagnostic tools. Our findings are comparable to Stringer, with further support from other studies like Frountzas et al. and Mussab et al. [16, 21-22]. However, differences in cut-offs, patient groups, and settings across studies may explain why our findings are slightly different. These findings are opposite to the findings of Favara et al., who reported high accuracy for the RIPASA score [14]. In our study, overall diagnostic accuracy was similar for both scores, with Alvarado showing a slight edge 89% vs. 88%. This finding is consistent with Mussab et al., though most meta-analyses report RIPASA as having higher sensitivity and overall accuracy, while Alvarado tends to be more specific, as mentioned in Favara et al.’s study [14, 22]. In our study, when the ROC curve was observed, its AUC for RIPASA was slightly better than that for the Alvarado score, which is consistent with the findings in the meta-analysis of Chong et al. and Favara et al. [3,14]. In conclusion, both Alvarado and RIPASA scores are effective for diagnosing acute appendicitis. Alvarado may be slightly more accurate overall, whereas RIPASA provides better confirmation when a higher value is required. Using these scoring systems in combination, which is based on clinical and blood investigations, can improve patient selection for surgery.

However, this study has some limitations. It was conducted at a single center with a relatively small sample size (100) compared to Mussab et al. (153), which may limit generalizability [16]. In addition, intraoperative diagnosis alone may not fully account for atypical cases. Further multicenter studies with larger cohorts and inclusion of imaging modalities are recommended to validate these findings. Furthermore, studies suggest using histopathological confirmation of acute appendicitis diagnosis.

## Conclusions

The Alvarado score demonstrated slightly higher overall diagnostic accuracy and sensitivity, while the RIPASA score showed superior specificity and PPV. Both are reliable tools for diagnosing acute appendicitis, with RIPASA offering an advantage in reducing negative appendectomy rates in resource-limited settings and surgical decision-making.
